# Successful cloning of a superior buffalo bull

**DOI:** 10.1038/s41598-019-47909-8

**Published:** 2019-08-06

**Authors:** Naresh L. Selokar, Papori Sharma, Monika Saini, Suman Sheoran, Rasika Rajendran, Dharmendra Kumar, Rakesh Kumar Sharma, Rajender K. Motiani, Pradeep Kumar, A Jerome, Sudhir Khanna, Prem Singh Yadav

**Affiliations:** 1 Division of Animal Physiology and Reproduction, ICAR-Central Institute for Research on Buffaloes, Hisar, 125001 India; 2 Animal Farm Section, ICAR-Central Institute for Research on Buffaloes, Hisar, 125001 India; 3 Systems Biology Group, CSIR-Institute of Genomics and Integrative Biology, New Delhi, 110025 India; 4 Present Address: Department of Obstetrics and Gynecology, All India Institute of Medical Sciences, New Delhi, 110029 India; 5 Present Address: Laboratory of Calciomics and Systemic Pathophysiology, Regional Centre for Biotechnology, Faridabad, 121001 India

**Keywords:** Cloning, Reprogramming

## Abstract

Somatic cell nuclear transfer (SCNT) technology provides an opportunity to multiply superior animals that could speed up dissemination of favorable genes into the population. In the present study, we attempted to reproduce a superior breeding bull of Murrah buffalo, the best dairy breed of buffalo, using donor cells that were established from tail-skin biopsy and seminal plasma. We studied several parameters such as cell cycle stages, histone modifications (H3K9ac and H3K27me3) and expression of developmental genes in donor cells to determine their SCNT reprogramming potentials. We successfully produced the cloned bull from an embryo that was produced from the skin-derived cell. Growth, blood hematology, plasma biochemistries, and reproductive organs of the produced cloned bull were found normal. Subsequently, the bull was employed for semen production. Semen parameters such as CASA (Computer Assisted Semen Analysis) variables and *in vitro* fertilizing ability of sperms of the cloned bull were found similar to non-cloned bulls, including the donor bull. At present, we have 12 live healthy progenies that were produced using artificial insemination of frozen semen of the cloned bull, which indicate that the cloned bull is fertile and can be utilized in the buffalo breeding schemes. Taken together, we demonstrate that SCNT can be used to reproduce superior buffalo bulls.

## Introduction

Somatic cell nuclear transfer (SCNT) can contribute to genetic improvement of livestock species by supplying seed stock animals, particularly superior bulls, for breeding schemes^1^. Clones of superior bulls can continue to be used for breeding schemes if original bulls are unable to produce semen due to old age, infection, injury, and/or their limited frozen semen-doses are available in the semen bank^1^. For cloning of breeding bulls, any diploid cell of the body, including semen-derived somatic cells, can be used as nuclear donors^2^. Here, we used two donor cell types, namely skin-derived somatic cells (SkC) and semen-derived somatic cells (SeC) to clone a superior buffalo breeding bull. To foresee the reprogramming potential of SkC and SeC, we carried out several investigations such as cell cycle analysis of donor cells, there *in vitro* developmental competence and quality of produced SCNT embryos. Further, we evaluated the status of epigenetic histone markers like acetylation of H3 lysine 9 (H3K9ac) and trimethylation of H3 lysine 27 (H3K27me3), mRNA abundance of certain genes, and *in vivo* developmental competence of produced SCNT embryos.

Previous studies have reported that cloned cattle bulls have similar reproductive performance as their donors, and progenies of cloned bulls did not display any abnormalities^3–5^. Despite several births of buffalo clones worldwide^2^, no information is available on health and reproductive performance of any born cloned buffalo, which is essential if cloned bulls are aimed to be employed in the breeding schemes. Therefore, to determine the suitability of the cloned buffalo bulls for breeding schemes, we examined several postnatal parameters such as growth, hematology, blood plasma biochemistry, semen attributes, and fertility of the produced cloned bull.

## Results

### Culture and characterization of donor cells and distribution of their cell cycle stages

**Figure 1:**
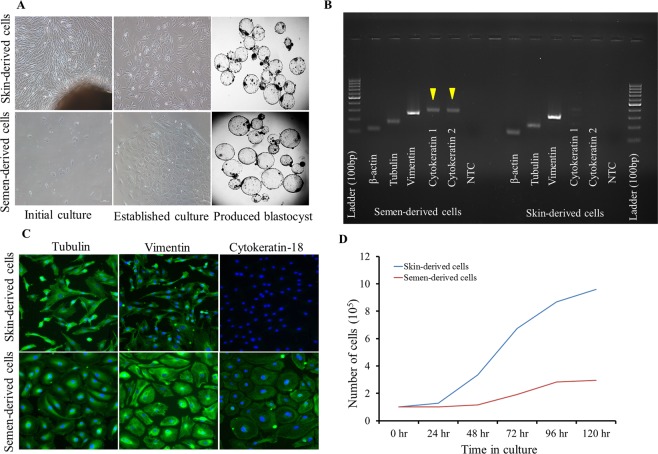
Culture and characterization of skin- and semen-derived somatic cells of the buffalo breeding bull. Skin-derived cells have spindle-shaped morphology; whereas, semen-derived cells have polygonal-shaped morphology and form a flat monolayer (**A**), both the type of cells were used to produce cloned embryos that developed to blastocysts (**A**). Skin-derived cells expressed vimentin, a fibroblast cell type marker; whereas, semen-derived cells expressed cytokeratin, an epithelial cell type marker, which were confirmed by mRNA expression using PCR (**B**) and immunofluorescence staining (**C**). Semen-derived epithelial cells have lower cell proliferation than that of skin-derived fibroblast cells (**D**). Cell images were captured at 100X total magnification; whereas, blastocyst images were captured at 40X total magnification.

**Figure 2:**
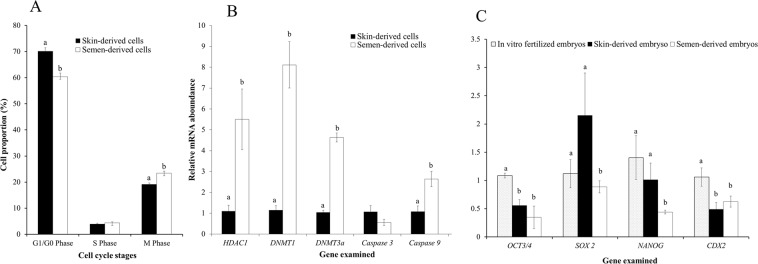
Distribution of cell cycle stages of donor cells, and mRNA expression of some developmental genes in donor cells and embryos. Somatic cells were cultured under identical culture conditions such as the same culture media, culture time, and culture surface. Cell cycle stages distribution of skin-derived somatic cells and semen-derived somatic cells (**A**), mRNA expression of epigenetic-related genes (*HDAC1, DNMT1, and DNMT3a*), apoptosis-related genes (*Caspase 3 and Caspase 9*) in both cell types (**B**). mRNA expression of pluripotency genes (*OCT 3/4, SOX2, NANOG, and CDX2*) in cloned blastocysts and IVF embryos (**C**). mRNA expression of the skin group was set to 1 in the case of somatic cells; whereas, the IVF embryo group was set to 1 for embryos. Bars with different superscript letters are significantly different (P < 0.05).

It is possible to culture somatic cells from seminal plasma, a non-invasive source of somatic cells^6^. In this study, we used a previously described method^6^ to culture somatic cells from ejaculated fresh semen of a superior breeding bull (Mu-4354). SeC appeared polygonal in shape and expressed cytokeratin-18, indicating that cultured cells belonged to the epithelial cell type; whereas, SkC belonged to the fibroblast cell type (Fig. 1A–C), which is in agreement with previous studies^6,7^. As reported earlier^6^, cell proliferation of SeC was lower than that of SkC (Fig. 1D). Before use of established somatic cells in SCNT experiments, we analyzed the distribution of cell cycle stages using flow cytometry. We observed that the higher distribution of SkC in G1/G0 cell cycle stage than that of SeC (70.1 ± 1.2 vs 60.3 ± 1.4); whereas, distribution of cells in the M stage was lower for SkC (19.1 ± 0.5 vs 23.4 ± 0.7, SkC vs SeC respectively) (Fig. 2A). And, no difference was observed for the distribution of cells in the S stage.

### Gene expression analysis in donor cells

DNMTs (DNA methyltransferases) and HDACs (histone deacetylase) are responsible for epigenetic modifications of donor cells. We analyzed the relative mRNA expression of *HDAC1*, *DNMT1*, *DNMT3a*, and *CASPASE3* and *CASPASE9* genes in donor cells. Similar to the previous report^6^, the mRNA abundance of *HDAC1* and *DNMT1* was found lower in SkC than that of SeC (Fig. 2B); also, the expression of *DNMT3a* and *CASPASE9* was observed lower in SkC than that of SeC. However, no difference was observed for *CASPASE3* expression.

### *In vitro* developmental competence of cloned embryos

*In vitro* developmental competence was evaluated in terms of cleavage and blastocyst rates, and the quality of developed blastocysts was evaluated by determining their total cell number and cell apoptotic index. We found similar cleavage and blastocyst rates of embryos that were produced from SkC and SeC (Table 1). Though, total cell number per blastocyst was similar among all groups, the cell apoptotic index was higher in blastocysts produced from SeC than that of SkC and IVF (Table 1), which indicate that the embryos produced from SkC were more healthy than those produced from SeC. Appropriate expression of transcription factors such as *OCT3/4*, *SOX2*, *NANOG*, and *CDX2* is required for efficient reprogramming following SCNT and these factors are considered as markers of pluripotency^8^. Here, we found that mRNA expression of *OCT3/4* and *CDX2* was lower in cloned blastocysts than that of IVF counterpart. However, the expression of *NANOG* and *SOX2* was comparable between blastocysts produced from SkC and IVF; whereas, it was lower in blastocysts produced from SeC (Fig. 2C).

**Table 1:** *In vitro* and *in vivo* development competence of cloned embryos of a superior buffalo bull.

Embryo type	Embryo cultured	*In vitro* development				*In vivo* development		
		Cleaved n (%)	Blastocysts n (%)	Total cell number	Apoptotic index	Recipient animals	Pregnancy	Live birth
SCNT skin-derived	175	157 (89.0 ± 1.5)	73 (40.4 ± 2.8)	282.2 ± 38.08	8.5 ± 1.9^a^	8	2	1
SCNT semen-derived	203	185 (90.5 ± 3.5)	70 (37.9 ± 8.5)	310.6 ± 12.7	11.2 ± 1.8^b^	7	0	0
IVF	340	189 (55.5)	52 (15.3)	210.6 ± 45.6	6.36 ± 1.9^a^	—	—	—

Data from 12 trails, values with different superscripts within the same column differ significantly (P < 0.05). *In vitro* fertilized (IVF) embryos produced using semen of donor bull as controls for cell apoptotic index analysis.

### Status of histone epigenetic markers

**Figure 3:**
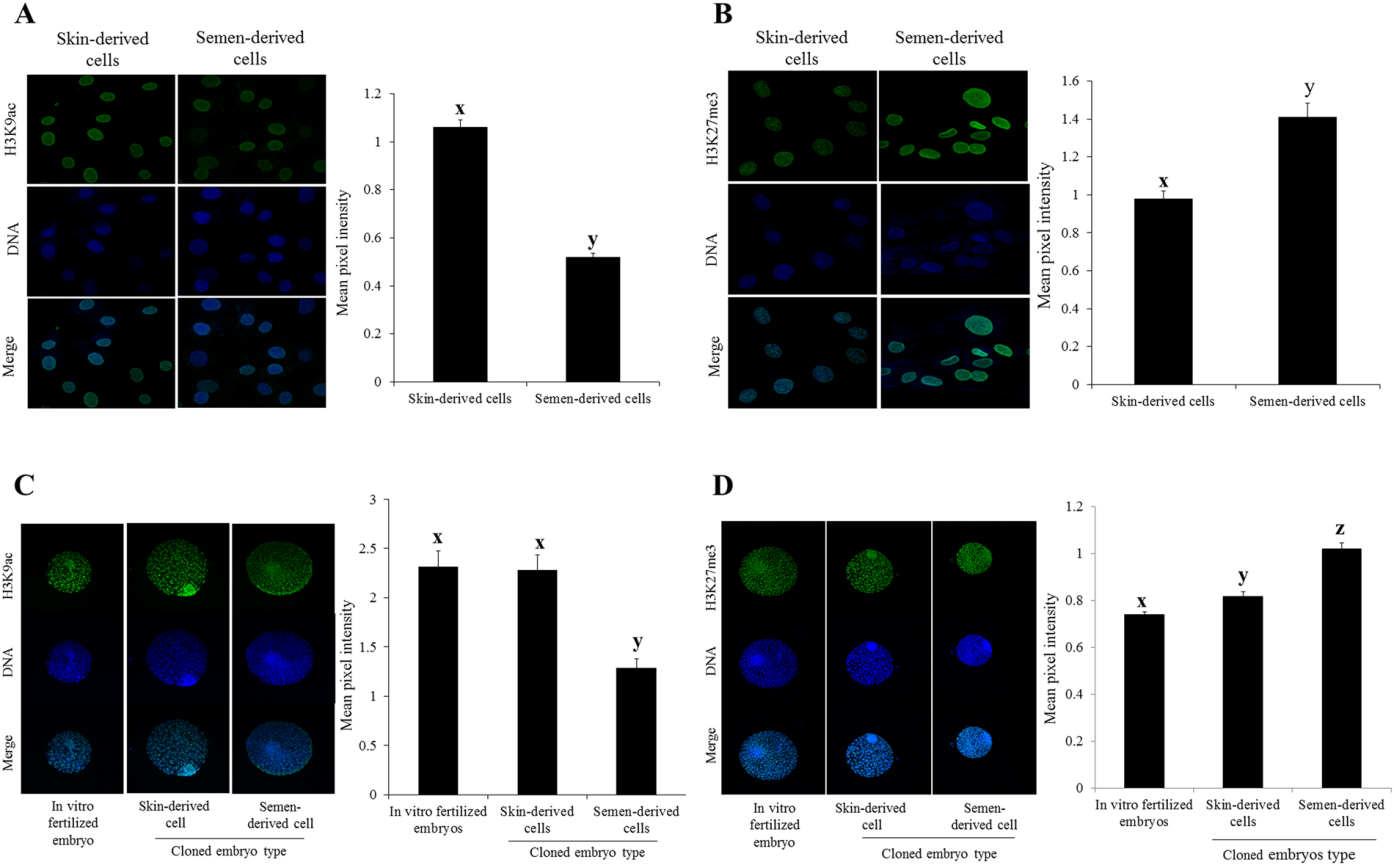
Epigenetic staining of somatic cells and their respective cloned embryos. Mean pixel intensity of histone H3 lysine 9 acetylation (H3K9ac) and Histone H3 lysine 27 trimethylation (H3K27me3) in somatic cells (**A**,**B**) and their respective cloned blastocysts (**C**,**D**), which was determined by the confocal microscopy. Somatic cell images were captured at 400X total magnification; whereas, blastocysts images were captured at 100X total magnification. Bars with different superscripts are significantly different (P < 0.01).

We studied two histone modification markers, namely H3K9ac and K3K27me3, which play an important role during early cloned embryonic development^9–11^. We observed that the levels of H3K9ac were significantly higher; whereas the levels of H3K27me3 were significantly lower in SkC than that of SeC (Fig. 3A,B). It was of further interest to investigate levels of these two epigenetic markers in cloned blastocysts when reprogramming had already completed and early differentiation process started. We observed that the level of H3K9ac was in the pattern of IVF blastocysts or cloned blastocysts of SkC greater than cloned blastocysts of SeC. Whereas, the level of H3K27me3 was in the pattern of IVF embryos less than cloned blastocysts of SkC less than cloned blastocysts of SeC (Fig. 3C,D). These results indicate that the level of these epigenetic markers maintained similar pattern from somatic cells to blastocysts following SCNT reprogramming. In addition, blastocysts produced from SkC were epigenetically more close to IVF blastocysts.

### Birth of cloned bull and its post-natal development

**Figure 4:**
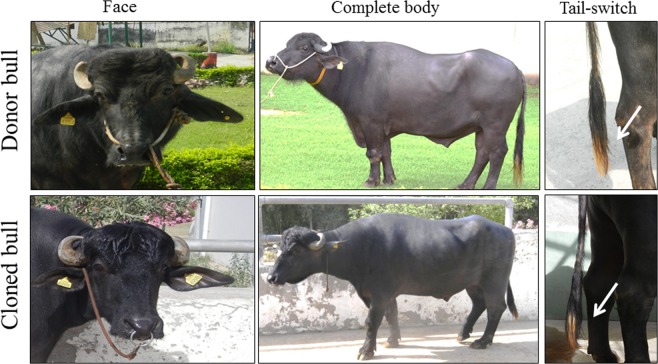
Donor bull and its cloned. Cloned bull has no physical abnormalities on the body, including face. White tail-switch color mark is identical to its donor bull (indicated by arrow).

To produce cloned bulls, we transferred one blastocyst stage embryo into the ipsilateral horn of each recipient buffalo. Eight and seven buffaloes were used as recipients to transfer blastocysts of SkC and SeC, respectively. Two recipients found pregnant following transfer of cloned blastocysts of SkC; whereas, no pregnancy was obtained with blastocysts of SeC (Table 1). Out of two positive pregnancies, one pregnancy completed full term and delivered the calf, with birth weight of 42 kg, through normal parturition; while, other pregnancy was aborted at the first trimester. DNA microsatellite based analysis confirmed the parentage of the born calf (Supplementary Table 1). We did not observe any physical and pathological abnormality in the cloned placenta. Further, no physical abnormalities in umbilicus, face, eyes, nostrils, and legs of the cloned calf were observed at birth. In addition, hematological and plasma biochemical indices of the cloned calf were within the normal reference ranges of buffalo (Supplementary Table 2). The cloned bull has no body structure deformities and has similar body mark at tail- switch which found in donor (Fig. 4). The born calf is now 40 months of age with body weight of 760 kg and has good health and producing good quality semen.

### Semen production and fertility of the cloned bull

Before employing to produce semen, we confirmed that the bull is free from physical abnormalities of reproductive organs, chromosomal abnormalities, and genetically transmitted diseases (factor XI deficiency syndrome, bovine leukocyte adhesion deficiency, and bovine citrullinemia) (data not shown). Following the screening and training, the cloned bull started donating semen at 19 months of age. We recorded multiple semen parameters such as ejaculated volume, sperm concentration, and mass sperm motility, which were found similar to other non-cloned bulls, including the donor bull (Table 2). In addition, computer assisted semen analysis (CASA) variables of the cloned bulls’ sperm were also found similar to that of non-cloned bulls (Table 3). At present, we have 10000 frozen semen doses of this cloned bull and semen production is ongoing.

**Table 2:** Fresh semen parameters of the cloned bull and non-cloned bulls, including donor bull.

Bull	Semen collections (n)	Ejaculated volume (ml)	Sperm concentration (x 10^6^ sperm/ml)	Mass motility (1–5 scale)	Progressive motility before freezing (%)	Post thaw- sperm motility (%)	Sperm abnormalities (%)	Acrosome integrity (%)
Cloned bull	10	2.4 ± 0.3	944.4 ± 130.6	3.4 ± 0.1	76.5 ± 1.0	54.5 ± 1.1	8.5 ± 0.2	94.6 ± 0.7
Donor bull	10	3.1 ± 0.2	1014.3 ± 74.3	3.68 ± 0.2	75.5 ± 0.5	50.5 ± 0.5	8.9 ± 0.2	91.3 ± 0.4
Other control bulls (n = 10)	10	2.7 ± 0.3	1115.0 ± 93.9	3.80 ± 0.2	77.5 ± 0.8	54.0 ± 1.2	9.7 ± 0.3	91.3 ± 0.5

Values are expressed as mean ± SEM. No differences were observed for pairs of means for any trait (P > 0.05).

**Table 3:** Computer-assisted semen analysis (CASA) variables of the cloned bull, donor bull and control bulls (n = 10).

Bull	VAP (µm/s)	VSL (µm/s)	VCL (µm/s)	ALH(µm)	BCF(Hz)	STR (%)	LIN (%)	TM (%)	PM (%)
Cloned bull	107.4 ± 4.2	76.6 ± 2.4	199.9 ± 8.5	8.1 ± 0.3	31.4 ± 0.9	72.8 ± 1.7	41.5 ± 1.2	60.8 ± 2.5	23.0 ± 2.4
Donor bull	109.0 ± 2.2	70.2 ± 2.3	220.8 ± 8.1	8.7 ± 0.3	30.1 ± 0.8	66.8 ± 1.9	35.3 ± 1.9	54.9 ± 2.6	17.4 ± 2.4
Other control bulls (n = 10)	102.9 ± 4.3	72.3 ± 2.5	193.0 ± 8.4	7.6 ± 0.3	32.1 ± 1.5	72.0 ± 2.1	40.6 ± 2.0	60.4 ± 1.8	21.6 ± 1.8

Data obtained from three to five independent replicates. VAP, average path velocity; VSL, straight linear velocity; VCL, curve linear velocity; ALH, average lateral head displacement; BCF, beat cross frequency; STR, straightness; LIN, linearity; TM, total motility; PM; progressive motility. Values are expressed as mean ± SEM. No differences were observed for pairs of means for any trait (P > 0.05).

To predict the suitability of this cloned bull for breeding schemes, we performed *in vitro* and *in vivo* fertilization studies using cloned bull semen. *In vitro* blastocyst production rate (18%) of cloned bull semen was comparable to the donor bull semen (16%). Following artificial insemination with frozen semen doses, the conception rate (55%) of cloned bull semen was comparable to non-cloned bulls, including donor bull (50–60%, conception rate recorded in our institute’s buffalo farm). The progenies of cloned bull (n = 12) are healthy and have normal growth. Based on the above findings, we conclude that the produced cloned bull is fertile and can be used for breeding schemes.

## Discussion

SCNT provides an opportunity to the animal breeders to restore superior bulls into breeding schemes; which otherwise could not produce semen due to old age/injury or a very limited number of their semen doses are available in the semen bank. Produced clones of superior bulls can help to extend the impact of an elite animal’s genetic by increasing supply of semen and/or embryos of the same genetics. In this study, we successfully cloned the superior breeding bull of the Murrah buffalo. The produced cloned bull has normal growth, health, and reproductive performance. Therefore, this cloned bull can be used in buffalo breeding schemes.

To clone the bull, we cultured somatic cells from two sources, skin-tissue and seminal plasma. In agreement with previous reports^6,7^, we confirmed that SeC belonged to an epithelial cell type and their proliferation rate was slower than that of SkC (Fig. 1). Previous studies in buffalo reported that use of G1/G0 synchronized fibroblast cells as donors improved the *in vitro* developmental competence of cloned embryos^12^. In our study, we observed that SkC were more synchronized in G0/G1 stage of the cell cycle than SeC (Fig. 2A); however, G0/G1 stage synchronization did not improved blastocyst production rate (Table 1). Several previous studies also suggested that donor cells at any cycle stage can be effectively reprogrammed and can produce cloned animals (reviewed by^13^).

Abnormal epigenetic reprogramming during early embryonic development is considered as a main cause of low conception rate following the transfer of cloned embryos and could also lead developmental defects in cloned animals^9,10^. We examined the pattern of epigenetic markers, namely H3K9ac and H3K27me3, of donor cells and attempted to understand if they translate to their corresponding embryos or altered following SCNT reprogramming. We observed that the pattern of donor cells’ epigenetic markers was similar to their corresponding embryos. However, the levels in cloned embryos of SkC were more close to IVF embryos than that of SeC embryos. In addition to epigenetic markers, the mRNA abundance of two pluripotency genes (*NANOG* and *SOX2*) was similar between cloned embryos of SkC and IVF embryos, which were found further down-regulated in cloned SeC embryos (Fig. 2C). However, the expression of *OCT3/4* and *CDX2* was down-regulated in cloned embryos of both cell types in compared to IVF embryos. The epigenetic and gene expression studies in donor cells indicate that donor cell source has significant effect on SCNT reprogramming, which is in agreement with previous report^6^.

Production of healthy progeny is the ultimate test to determine the reprogramming potential of any donor cell type. In this study, we produced the healthy calf following the transfer of cloned blastocyst of SkC. It has been reviewed that the postnatal mortality is very high in buffalo clones^14^. Therefore, following the birth of the cloned calf, we have taken special care and examined several post-natal parameters to monitor its health status. The calf’s hematological and plasma biochemical indices were normal, which is in agreement with the previous reports in cattle^15^. In addition, we followed bull’s screening procedures that are required before employing bulls for semen production including chromosomal analysis, genetic disease testing and health of reproductive organs. After the screening, the cloned bull was employed for semen production. Before this study, it was not clear whether cloned buffalo bull(s) are reproductively as competent as their donor(s), since no information was available on the reproductive performance of cloned buffalo bulls. Therefore, we have studied the reproduction potential of produced cloned bull.

The reproductive potential of the cloned bull can be evaluated by studying sperm parameters and by conducting artificial inseminations in the farm and/or production of IVF embryos in the laboratory^16^. In agreement with previous reports on *in vitro* semen attributes of cloned bulls of cattle^4,5^, bucks^17^, and boars^18^, we are reporting for the first time that several *in vitro* sperm parameters, including CASA variables and sperm fertility rate, of the cloned buffalo bull were similar to non-cloned bulls including its donor (Tables 2, 3). However, *in vitro* sperm parameters and IVF results do not always predict the actual fertility of bull, since sperms are complex in nature and technical variability in IVF protocols cannot be neglected^16^. Artificial insemination (AI) is the most accurate method to determine actual fertility of cloned bull. We found that the fertility rate following AI with the cloned bull was comparable to donor bull. At present, we have 12 live normal progenies (age ranges from 3–4 months) of the cloned bull with normal birth weights and normal growth (data not shown). We also established more pregnancies from the cloned bull semen, which are continuing. In addition to this cloned Murrah bull, we also cloned breeding bull of Assamese buffalo, which is now 14 months of age and showing normal health parameters (unpublished work).

Taken together, we conclude that SCNT can reproduce superior buffalo bulls that can be utilized in breeding schemes. Going forward, further large-scale studies and extensive data collection are required to confirm that cloned buffalo bulls and their progenies are normal, healthy and reproductively as competent as the progenies of non-cloned bulls.

## Materials and Methods

### Animal ethics

All experiments, including animal experiments, were conducted as per relevant guidelines and regulations laid down by the Institute Animal Ethics Committee, ICAR-Central Institute for Research on Buffaloes (CIRB), Hisar, India. This study was approved by the Institute Animal Ethics Committee, CIRB, India, and efforts were made to minimize animals’ pain and suffering, and to reduce the number of buffaloes required to complete this study. In this study, we select the superior breeding bull (Mu-4354) of Murrah buffalo that has employed in the national buffalo improvement scheme. After the birth, the cloned bull has been rearing under identical farm conditions and same food diet with non-cloned bulls at our institute’s buffalo semen production center.

### Cell culture

To culture skin-derived cells, the skin tissue biopsy from the ventral side of tail origin was aseptically collected with the help of a notcher and collected tissue biopsy was processed to culture somatic cells as previously described^19^. To culture somatic cells from the semen, freshly ejaculated semen was processed to culture somatic cells as previously described^6^. Cell type characterization and *in vitro* growth of cultured cells were determined as previously described^6^. The established cells (1 × 10^5^ cells/ml) at passage 2–3 were subjected to slow freezing at the cooling rate of 1.0 °C/ min to −80 °C in DMEM/F12 medium having 10% dimethyl sulphoxide (DMSO) and 20% fetal bovine serum (FBS), after which the cells were stored in liquid nitrogen for future use. Whenever needed, cells were thawed and cultured. Both cells types were maintained under identical culture conditions (similar culture surface and culture medium) and were used in different experiments at passage 5–10.

### Cell cycle analysis

Total confluent cells from T-25 cm^2^ flasks were dissociated using 0.25% trypsin and fixed in chilled 70% ethanol for overnight and stored at −20 °C freezer until analysis. For cell cycle analysis, fixed cells were washed with Dulbecco’s phosphate-buffered saline (DPBS) and stained with DNA binding solution (10 µg/mL propidium Iodide, 0.1 mg/mL RNase A and 0.1% Triton X-100) for 10 min. Subsequently, cells were transferred in DPBS, and cell cycle analysis was performed using a 488-nm laser line for excitation with the same algorithm for all samples in the BD FACS LSR-II (Becton-Dickinson, Rutherford, NJ, USA). For each cell type, 10000 cells were analyzed to determine the proportion of cells in G0/G1, S, and G2/M stage using the Flowjo Cycle Analysis software (Tree Star Inc., Ashland, OR). Three independent flow runs were performed for each cell type.

### Cloned embryo production and their quality assessment

Cloned embryos were produced according to the handmade cloning method, which was previously described by us^19^. For the production of *in vitro* fertilized (IVF) embryos, immature oocytes obtained from slaughterhouse ovaries were subjected to maturation followed by *in vitro* fertilization using frozen semen of the donor bull (Mu-4354) and the cloned bull, and fertilized zygotes were cultured as previously described^20^. Total cell number (TCN) and level of apoptosis in day 8 blastocysts (cloned and IVF) were determined by TUNEL staining as previously described^6^. For ideal comparison, both donor cell types (skin-derived and semen-derived cells) were used for the production of cloned embryos in the same SCNT experiments.

### Gene expression analysis

RNA was isolated from 1 × 10^5^ cells or blastocysts (n = 3 to 4) from three different replicates using the RNAqueous micro kit (Cat no # AM1931, Ambion, Austin, TX, USA) and subsequently converted to cDNA using superscript reverse transcriptase III kit (Cat no # 18080-051, Invitrogen). Real-time PCR based gene expression analysis was performed using the optimized primers (Supplementary Table 3) on a step one plus real-time system (Applied Biosystems, Foster City, CA, USA) with PowerUp™ SYBR® green master mix (Cat no # A25742, Invitrogen) at the following thermal cycling conditions: 50 °C for 2 min, 95 °C for 2 min, followed by 40 PCR cycles of 95 °C for 15 sec, 58 °C for 15 sec, and 72 °C for 15 sec. For ensuring specific gene amplification, a melting curve was determined for each gene and 2% agarose electrophoresis was carried out to determine the length of the amplified PCR products. β-actin mRNA was employed as an internal control for the analysis of relative transcript levels of each gene and in negative controls (H_2_O replaced with cDNA in the reaction tubes). For comparison, the average expression levels of each gene from skin-derived cells were set to 1 for analysis in somatic cells, whereas expression levels from IVF blastocysts produced from donor bull semen were set to 1 for analysis in blastocyst stage embryos.

### Epigenetic staining

Donor somatic cells (1 × 10^5^ cells grown on 22 mm × 22 mm glass coverslips) and blastocysts (cloned and IVF) were washed in DPBS and fixed with 4% formaldehyde in DPBS for 1 h and then permeabilized with 0.1% Triton X-100 in DPBS for 30 min, followed by 1 h blocking with 3% BSA solution in DPBS. Subsequently, somatic cells and blastocysts were incubated with primary antibodies; anti-H3K9ac (1:1000, Sigma) and anti-H3K27me3 (1:1000, Millipore), diluted in 3% BSA solution at 4 °C for the overnight period. After 5 washing with DPBS containing 0.1% Triton X-100 (DPBST) at 3 min intervals, somatic cells and blastocysts were incubated with FITC-conjugated goat anti-rabbit secondary antibody diluted 1:700 in DPBS for 90 min. Five times of washing were given with DPBST, and nuclei were counterstained with H33342 (10 µg/ml) and rinsed in DPBS. Stained somatic cells and blastocysts were then mounted on glass slides in mounting medium (2.5% DABCO in glycerol). Blastocysts images were captured using the laser confocal microscope (Leica TCS SP8) with a Plan Apo 10 ×/ 0.4 NA objective having excitation wavelengths of 488 and 561 nm, and somatic cells were imaged with 100 ×/ 1.4 NA oil immersion objective at above-mentioned wavelengths. Collection of each channel signals was done sequentially. For each wavelength, serial optical sections (Z-stack) were collected at 2.5 μm intervals through the blastocysts specimens and 0.3 μm intervals through the somatic cells specimens, with scan averaging and slow scan speed. Similar detector gain, amplifier offset, and pinhole parameters were used during all imaging experiments. Z-stacks were merged to produce two-dimensional images which depict the staining patterns and total intensities of all nuclei. The Leica LAX-1.8.1.13 software was used for image acquisition and quantitative measurements of the mean pixel intensity emitted by each individual nucleus. At least five independent images of somatic cells (8–10 nuclei from each image) or five blastocysts (~80–100 nuclei from each blastocyst) were analyzed for each epigenetic marker.

### Embryo transfer

Cyclic buffaloes having functional corpus luteum and 10–12 mm dominant follicles (observed through ultrasonography) were treated with PGF2α analog (Cloprostenol, 500 µg) intramuscularly. Those exhibited estrus within 48–96 h after PGF2α analog injection were selected as recipients for cloned embryo transfer (ET). At day 8, one blastocyst was transferred into the ipsilateral uterine horn of recipient. Pregnancies were confirmed by transrectal ultrasonography at day 30 and were reconfirmed again at day 60 and 90 after ET. The CALF lab, National Dairy Developmental Board (NDDB), India, has conducted the parentage confirmation of the born calf using microsatellite markers. The whole placenta was collected and fixed in 10% neutral buffered formalin. Histology of placenta was performed using standard pathology methods.

### Blood hematology

Blood samples (n = 2) from the cloned calf at 12 and 18 months of age were collected and immediately processed for the determination of hematological variables using an automatic veterinary hematological analyzer (VetScan HM5, Model No: 250735; M/s Abaxis, Pvt. Ltd, USA).

### Blood plasma biochemistry

Blood samples (n = 2) from the cloned calf at 12 and 18 months of age were collected in heparinized vacutainer tubes. Collected blood samples were centrifuged at 100 × g for 5 min for separation of plasma. Biochemical parameters of plasma were estimated using automatic biochemical analyzer (Coralyzer 200, Tulip Diagnostics, Pvt. Ltd. India).

### Chromosomal analysis and genetic diseases analysis

Chromosomal spreads from the fresh blood sample of the cloned calf were prepared and analyzed for chromosomal abnormalities as previously described^21^. In addition, the cloned calf was screened for genetic disorders, namely bovine leukocyte adhesion deficiency, bovine citrullinemia and factor XI deficiency, by the CALF lab, NDDB, India.

### Ultrasonography of testes

The diagnostic ultrasound scanner (Vision 200, Model SSA 320A, Toshiba, Japan) with a 7.0 MHz micro-convex multi-frequency transducer was used to scan testes. Coupling gel was applied on the surface of the testis and on the face of the transducer. During scanning, the moderate pressure was used to press the transducer against the skin on the caudal scrotal surface; whereas, for an anterior surface, the testis was supported by hands. Each testis was examined separately with the transducer placed vertical and parallel to the long axis of the testis and was aligned so that the mediastinum testis was readily apparent as a continuous band across the image. Ultrasound images of each testis were captured and saved and evaluated for any echogenicity of testicular parenchyma and for pathological lesions.

### Analysis of semen of cloned bull

The cloned bull started donating semen at the age of 19 months; the semen was collected using an artificial vagina method. Various parameters such as volume and sperm concentration of freshly ejaculated semen were recorded. Mass sperm motility score of freshly ejaculated semen were recorded on the scale of 0–5; 0 corresponds to no swirl, 1 corresponds very slow individual swirl (generalized oscillation of individual sperm only), 2 corresponds to very slow distinct swirl, 3 corresponds slow distinct swirl, 4 corresponds moderately fast distinct swirl, and 5 corresponds to rapid swirl. Following the primary observations, semen was diluted in egg yolk based extender and was loaded into 0.25 mL plastic straws (20 million sperm/straw), and then filled straws were frozen using a programmed freezer (Mini Digi-cool, IMV Technologies, L’Aigle, France) as previously described^22^ and subsequently stored in liquid nitrogen for long term. Percentage of progressively motile thawed sperm was visually estimated. In addition, the multiple sperm attributes such as morphometric analysis, viability, acrosome membrane integrity, and computer-assisted sperm analyzer (CASA) indices were determined in freshly ejaculated semen samples as previously described^23,24^. All microscopic examinations were done by a single operator to minimize variability in the results.

### Fertility of cloned bull

IVF was done as described above. For artificial insemination (AI), the frozen-thawed semen from the cloned bull was used to inseminate 49 buffaloes; whereas, 60 buffaloes were inseminated with semen of its donor bull. Transrectal ultrasonography was done at day 30 after AI to detect pregnancies and positive pregnancies were reconfirmed again at day 60.

### Statistical analysis

All data are expressed as means ± SEM and were analyzed using the Student’s t-test or ANOVA. The manuscript data were tested for normality and homogeneity of variances using SPSS 20 software (IBM SPSS Statistics for Windows, Version 20.0, IBM Corp Armonk, NY), and the log transformation was performed on the data which are not distributed normally before performing t-test or ANOVA. Differences between two groups were analyzed by Student’s t-test and for comparisons among more than two groups, one-way ANOVA followed by the Fisher LSD post hoc test was used. Differences were considered significant if the p-value was <0.05 (a, b & c) or <0.01 (x, y and z).

## Supplementary information

Supplementary information
