# Supplementary material for: Successful cloning of a superior buffalo bull
Source: Sci Rep. 2019 Aug 6;9:11366. doi: 10.1038/s41598-019-47909-8 (PMC6684639; doi:10.1038/s41598-019-47909-8)
Supplement: Supplementary file 1 — Supplementary information [file 41598_2019_47909_MOESM1_ESM.pdf]

Successful cloning of a superior buffalo bull

Naresh L. Selokar[a], Papori Sharma[a], Monika Saini[a*], Suman Sheoran[b], Rasika Rajendran[a], Dharmendra Kumar[a], Rakesh Kumar Sharma[a], Rajender K. Motiani[c#], Pradeep Kumar[b], Jerome A[a], Sudhir Khanna[c] and Prem Singh Yadav[a]

[a]Division of Animal Physiology and Reproduction, [b]Semen Freezing Laboratory, and [d]Buffalo farm of ICAR-Central Institute for Research on Buffaloes, Hisar 125001, India

[c]Systems Biology Group, CSIR-Institute of Genomics and Integrative Biology, New Delhi 110025, India.

Present address:

[*]Department of Obstetrics and Gynecology, All India Institute of Medical Sciences, New Delhi110029, India.

[#]Laboratory of Calciomics and Systemic Pathophysiology, Regional Centre for Biotechnology, Faridabad 121001, India.

Correspondence and requests for materials should be addressed to PSY (psycirb@gmail.com) or NLS (selokarnareshlalaji@gmail.com).

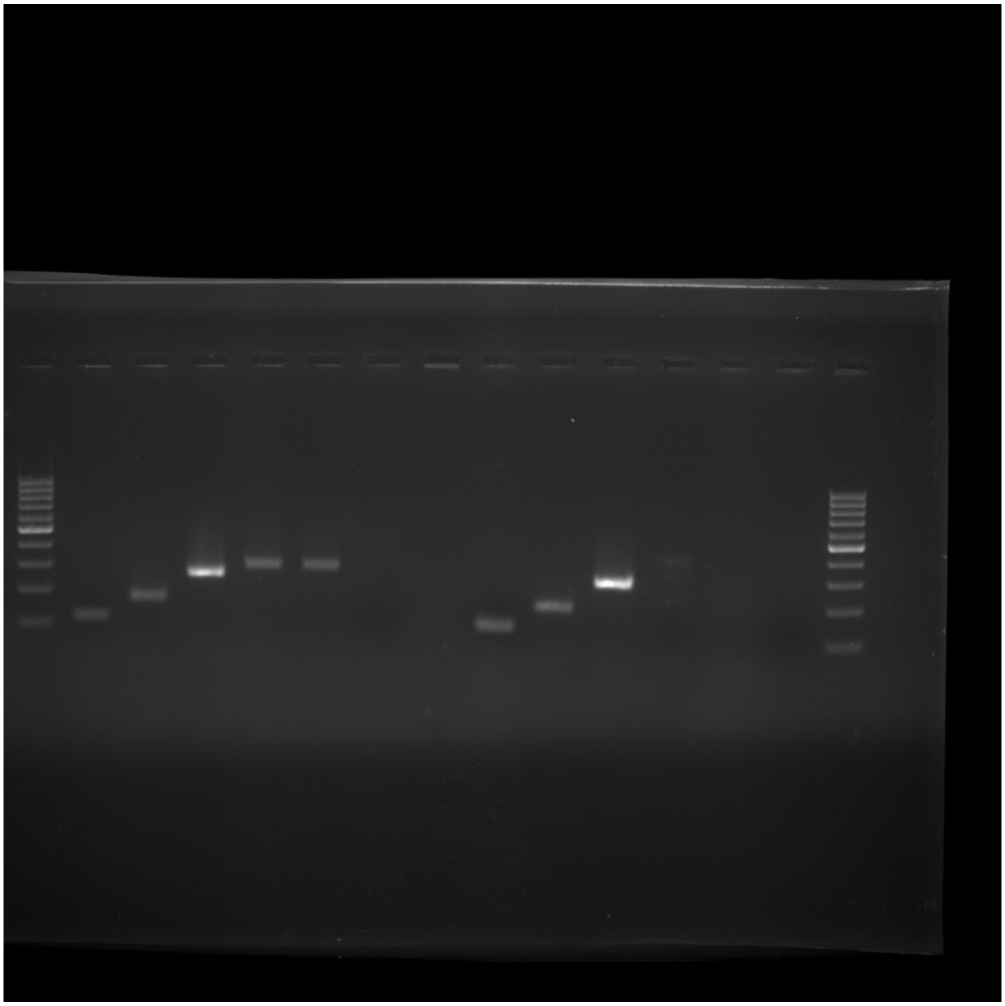

The original gel photo of figure 1B: Skin-derived cells expressed vimentin only, a fibroblast cell type marker; whereas, semen-derived cells expressed cytokeratin, an epithelial cell type marker, which were confirmed by mRNA expression using PCR.

Supplementary table 1: Parentage verification of cloned calf by microsatellite based analysis

Sr. No.	Microsatellite Marker	Cloned bull		Donor bull		Recipient buffalo	
		**Allele 1**	**Allele 2**	**Allele 1**	**Allele 2**	**Allele 1**	**Allele 2**
1	Marker1	159	165	159	165	155	155
2	Marker2	110	112	110	112	110	110
3	Marker3	254	254	254	254	270	270
4	Marker4	202	202	202	202	220	220
5	Marker5	127	137	127	137	129	137
6	Marker6	80	86	80	86	86	86
7	Marker7	250	254	250	254	256	256
8	Marker8	207	207	205	207	205	205
9	Marker9	209	209	209	209	211	211
10	Marker10	126	138	126	138	116	126
11	Marker11	88	102	88	102	102	102
12	Marker12	132	136	132	136	120	134
13	Marker13	80	82	80	82	82	86

Valves represent PCR product size of microsatellite markers.

Supplementary table 2: Hematological and biochemical indices of the cloned calf

Parameter	Cloned bull		Reference values for buffalo
Hematological indices			
	at 12-month of age	at 18-month of age	
Hg (g %)	12.5	13.0	8-15
TEC ($\times 10^{12}$/L)	8.68	10.75	5.00-10.00
TLC ($\times 10^{9}$/L)	8.05	11.61	4.00-12.00
Lymphocyte (%)	60	41	45.00-75.00
Monocyte (%)	02	04	2.00-7.00
Neutrophil (%)	38	55	15.00-55.00
Eosinophil (%)	2.3	0	0.00-20.00
Basophil (%)	0.2	0	0.00-2.00
Blood plasma biochemistry indices			
	At 12-month of age	At 18-month of age	
Total protein (g/dl)	7.3	7.8	6.0-14.2
Cholesterol (mg/dl)	101	79.7	80-120
Triglyceride (mg/dl)	80.8	39	46-52[$]
Urea (mg/dl)	31.9	24.8	20-45
LDH (U/L)	706.6	1915	692-1445
AST (U/L)	50.9	83.5	78.0-132.0
ALT (U/L)	38.2	57.8	11.0-40.0

Hemoglobin (Hg), Total erythrocyte count (TEC), Total leukocyte count (TLC), Lactate dehydrogenase (LDH), Aspartate aminotransferase (AST), Alanine aminotransferase (ALT). Reference valves for hematological indices of buffalo are suggested by Weiss, D.J. & Wardrop, K.J. Schalm's Veterinary Hematology, 6th Edition, pp: 927-930, blood plasma reference values of buffalo are suggested by Kaneko, J.J. Harvey, J.W. & Bruss, M.L. Clinical Biochemistry of Domestic Animals. 5th ed. San Diego: Academic Press (1997), [$]based on Kumar, S. *et al.* Hemato-biochemical and hormonal profiles in post-partum water buffaloes (*Bubalus bubalis*). *Vet World* **8(4)**, 512-551 (2015).

Supplementary table 3: Primers used for gene expression analysis

**Gene**	**Sequence**	**Product size**	**Acc. No**
HDAC1	F-ATCGGTTAGGTTGCTTCAATCTG<br>R- GTTGTATGGAAGCTCATTAGGGA	168	BT030718.1
DNMT1	F-CTCAGAAGGGAGATGTGGAG<br>R-TAGTAGTCACAGTAGCTGAGGA	138	NM_182651.2
DNMT3a	F- GTGCTGTCTCTATTCGATGG<br>R- CCATTCCTGGATATGCTTCTG	188	NM_001206502.1
Caspase 3	F- TGGTATTGAGACAGACAGTGG<br>R-AGCATCTCACAAAGAAGCCTG	158	NM_001077840.1
Caspase 9	F-GTGTCCTACTCCACCTTCCC<br>R-TGAAACAGCATTAGCGACCC	150	NM_001205504
CDK2	F-CACCGAGACCTTAAACCTCAG<br>R-GTACCACAGAGTCACCACCT	132	NM_001014934
CDK3	F-AGATCAGGCTGGATTTGGAG<br>R-AGTCCATGTTACTTCTTCAGGTC	177	NM_001099178
CDK4	F-GAGCATCCCAATGTTGTCAG<br>R-GTGCCTTGTCCAGATATGTC	124	NM_001037594
OCT4	F-CGCCCTATGACTTGTGTGGA<br>R-TCGGCTCCAGCTTCTCCT T	201	GU997625.1
NANOG	F-CCGAAGCATCCAACTCTAGG<br>R-GAGACAGTGTCCGTGTCGAG	100	NM_001025344.1
SOX2	F-ATGGGCTCGGTGGTGAAGT<br>R- GCTCTGGTAGTGCTGGGACA	183	GQ853881.1
CDX2	F-GAGAAGGAGTTTCACTACAGTCG<br>R-TTCCTTTCCTTTGCTCTGCG	124	AT00000002378
B-ACTIN	F-ACCACACCTTCTACAACGAG<br>R-GAACATGATCTGGGTCATCTTC	112	NM_173979.3
